# Supplementary material for: Spatiotemporal observation of quantum crystallization of electrons
Source: Nat Commun. 2023 Sep 26;14:6011. doi: 10.1038/s41467-023-41731-7 (PMC10522630; doi:10.1038/s41467-023-41731-7)
Supplement: Supplementary file 1 — Supplementary information [file 41467_2023_41731_MOESM1_ESM.pdf]

Supplementary Information for

Spatiotemporal observation of quantum crystallization of electrons

Hideaki Murase[1], Shunto Arai[1,2], Tatsuo Hasegawa[1], Kazuya Miyagawa[1] and Kazushi Kanoda[1,3,4,5]*

[1]*Department of Applied Physics, University of Tokyo, Bunkyo-ku, Tokyo 113-8656, Japan.*
[2] *Research Center for Macromolecules and Biomaterials, National Institute for Materials Science (NIMS), Tsukuba, Ibaraki 305-0044, Japan.*
[3]*Max Planck Institute for Solid State Research, Heisenbergstrasse 1, 70569 Stuttgart, Germany*
[4]*Physics Institute, University of Stuttgart, Pfaffenwaldring 57, 70569 Stuttgart, Germany*
[5]*Department of Advanced Materials Science, University of Tokyo; Kashiwanoha 5-1-5, Kashiwa 277-8561, Japan*

*Corresponding author. Email: k.kanoda@fkf.mpg.de

Supplementary Note 1. Analysis of the Raman spectra

To characterize the electronic crystallization over time in θ-RbZn, we pursued the time evolution of the Raman spectrum, which was decomposed into supercooled liquid/glass (SCL/CG) and charge order (CO) components; the time evolution of the CO fraction was examined at each temperature studied. Supplementary Figure 1 shows the basis spectra of SCL/CG and CO at each temperature used for the analyses shown in Fig. 2 in the main text. $I^{\mathrm{CG}}(\nu)$ is the Raman spectrum of SCL/CG measured immediately after rapid cooling to each temperature, and $I^{\mathrm{CO}}(\nu)$ is the Raman spectrum of CO measured after crystallization is complete. In the measurements, a background trend that inevitably arises from stray light was superposed on the Raman spectra. As it is well approximated by the linear trend ($a\nu + b$) in Supplementary Fig. 2, we determined a and b by its fit to the experimental baseline outside the peak(s) (from 1300 cm^{-1} to 1400 cm^{-1} and from 1550 cm^{-1} to 1600 cm^{-1}). Then, the Raman spectra $I(\nu、t)$ during crystallization are expressed as:

$$I(\nu、t) = A^{\mathrm{CO}}(t)I^{\mathrm{CO}}(\nu) + A^{\mathrm{CG}}(t)I^{\mathrm{CG}}(\nu) + (a\nu + b), \qquad (1)$$

where $A^{\mathrm{CO}}(t)$ and $A^{\mathrm{CG}}(t)$ are the spectral weights of CO and SCL/CG at time t. The fraction of CO is evaluated by $\phi(t) = A^{\mathrm{CO}}(t)/(A^{\mathrm{CO}}(t) + A^{\mathrm{CG}}(t))$. Supplementary Figure 2 shows examples of the fitting at 198 K and 154 K. Figure 2a and Supplementary Fig. 1 show the spectra after subtracting the background trend $(a\nu + b)$.

Supplementary Note 2. Raman imaging

Supplementary Figure 3 shows all the contour plots of ϕ measured during the isothermal crystallization of electrons at T_{q} = 195 K and 155 K; the contour plots in Fig. 3 in the main text are representative plots. The time intervals are 149 s for the measurements at 195 K (Supplementary Fig. 3a) and 63 s for those at 155 K (Supplementary Fig. 3b). The spatial resolution is 6.5 μm.

Supplementary Note 3. Observation of crystallization in large areas

In Figs. 3a and 3b, the observation area is limited to 65×130 μm^2 and 65×65 μm^2, respectively, to obtain the high spatial resolution (6.5 μm) in the present apparatus. To ensure that the present observation is not specific to particular regions such as the vicinity of the domain boundaries, we also performed Raman imaging experiments for a larger area of 124.8×416 μm^2 by reducing the spatial resolution to 10.4 μm at 198 K (Supplementary Fig. 4a) and 150 K (Supplementary Fig. 4b). As seen in Supplementary Figs. 4a and 4b, the results are essentially the same as shown in Figs. 3a and 3b, respectively, confirming that the observed features are not dependent of the observation area.

Supplementary Note 4. About the evolution of Raman image while taking one image at high temperatures

Here, we quantitatively estimate the time evolution of the Raman image while taking one image. From the analysis (Fig. 4a) of the time evolution of the Raman image (Fig. 3a), we know that the growth speed of the CO domain is 40 nm/s at 195 K. On the other hand, it takes 149 s to obtain one image. From these values, the growth length of the CO domain while taking one image is 6.0 μm, which is smaller than the spatial resolution, 6.5 μm. The change of the Raman image while imaging is, therefore, negligible.

Supplementary Note 5. Origin of the mottled colours in Fig. 3b.

The images in Fig. 3b show spatially mottled colours. To characterize the pixel-by-pixel variation of the experimental ϕ values, we calculated the variance of ϕ, σ^2, for the data in Fig. 3b and it is plotted as a function of the averaged ϕ value, $\langle\phi\rangle$, in Supplementary Fig. 5a. Conceivable origins of the variance of ϕ are the spatial inhomogeneity in the distribution of the microcrystals and the noise in spectra.

First, we discuss the case of the inhomogeneity in the spatial distribution of microcrystals. For simplicity, we consider a site-percolation model where each site is either glass or crystal. When each site is crystal with probability, ϕ, the expected value and the variance of the volume fraction of the crystal are ϕ and $\sigma_{sp}^2 = \phi(1-\phi)/N$, respectively, where N is the number of sites in one pixel. Supplementary Fig. 5b shows the $\langle\phi\rangle$ dependence of σ_{sp} with N=10, 30, 100, which correspond to the sizes of the site, 2.1×2.1, 1.2×1.2 and 0.65×0.65 μm², respectively, in the present pixel size of 6.5× 6.5 μm². The σ_{sp} vanishes at $\langle\phi\rangle$=0 and 1, taking a peak at $\langle\phi\rangle$=0.5. The peak formation at $\langle\phi\rangle$=0.5 becomes suppressed as N increases. This $\langle\phi\rangle$ dependence of σ_{sp} is totally different from the experimental observation shown in Supplementary Fig. 5a.

Next, we discuss the case in which the noise in spectra causes pixel-by-pixel fluctuations of ϕ. In order to simulate this situation, we performed the following numerical calculations. First, we prepare ideal Raman spectra of CO and CG with small noise. The two spectra are added together so that the volume fraction of CO is equal to $\langle\phi\rangle$, and then the Gaussian noise is artificially added to it. In this way, 10000 spectra are calculated for each $\langle\phi\rangle$ and these spectra are fitted by the method described in the text. The variance of ϕ,σ_n^2 ,is calculated from the variation of the fitting results. The $\langle\phi\rangle$ dependence of σ_n is shown in Supplementary Fig. 5c, where σ_n increases monotonically with respect to $\langle\phi\rangle$. This reproduces the experimental results very well (Supplementary Fig. 5d). Thus, the main origin of the mottled colours in Fig. 3b is concluded to be the noise in the Raman spectra.

Supplementary Note 6. Directional dependence of the growth speed

In Fig. 4a, we presented the growth rate in a certain direction. In actuality, we examined the growth speed in different directions. Supplementary Figure 6 shows the angular dependence of the growth speed obtained by varying the angle of the arrow and fixing its origin in Fig. 4a, indicating that the growth speed has almost no angular dependence.

Supplementary Note 7. Charge fluctuation frequency used in the analysis of the CO growth rate

The previous study on the electrical resistance noise in θ-RbZn[1] succeeded in finding the lower and upper cut-off frequencies f_{c1} and f_{c2} of the charge fluctuations in the CL phase by analysing the noise spectrum with the distributed Lorentzian model. In the present analysis, as the charge fluctuation frequency, we adopted the geometric mean of f_{c1} and f_{c2}, $f_0=(f_{c1}f_{c2})^{1/2}$. However, in the SCL/CG phase, f_{c1} and f_{c2} and therefore f_0 were unknown because accurate noise spectroscopy was hindered by crystallization. Thus, we extrapolated the f_0 values known in the CL phase to the SCL/CG phase at lower temperatures, assuming the activation type of the temperature dependence:

$$f_0(T) = f' \exp\left(-\frac{E_g}{T}\right), \qquad (2)$$

where f' and E_g are determined to be $f' = 6.80 \times 10^{13}$ Hz and E_g = 5902 K by fitting Supplementary Eq. 2 to the data in Supplementary Fig. 7.

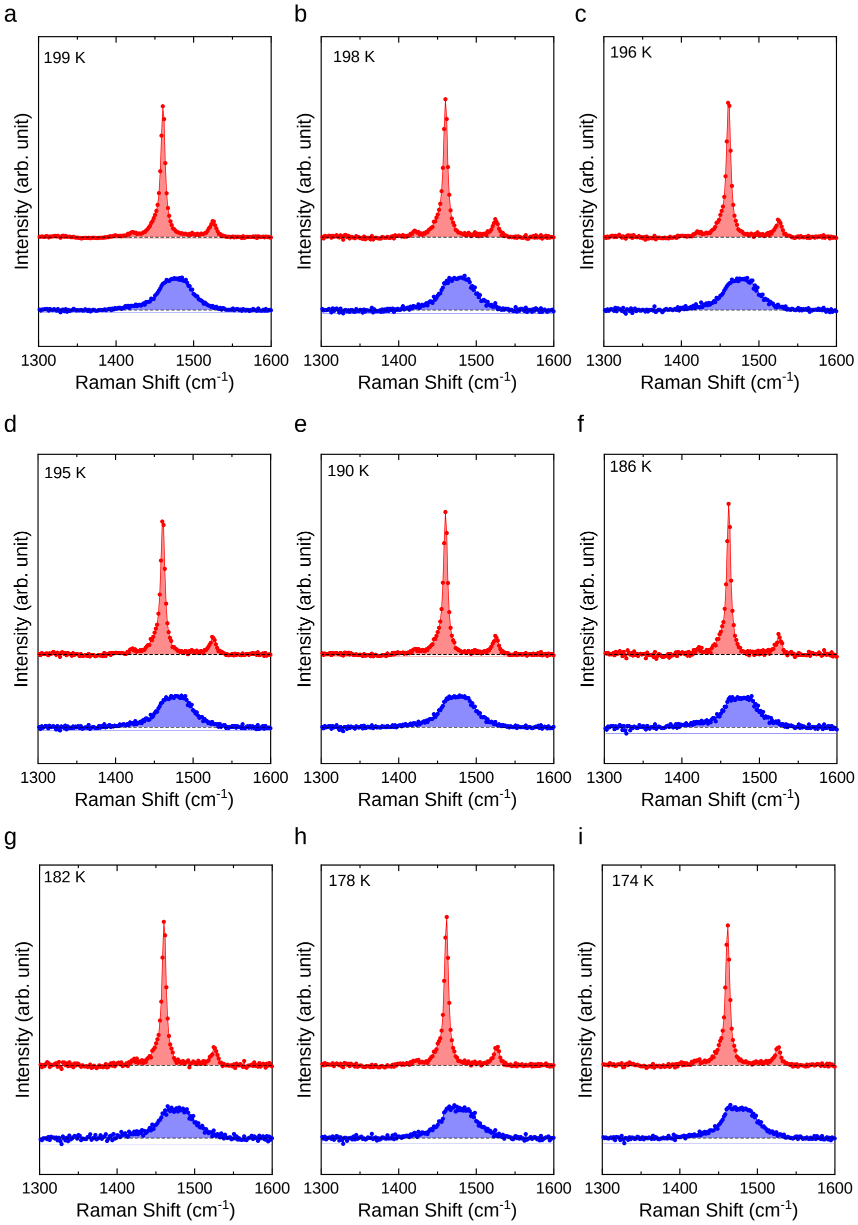

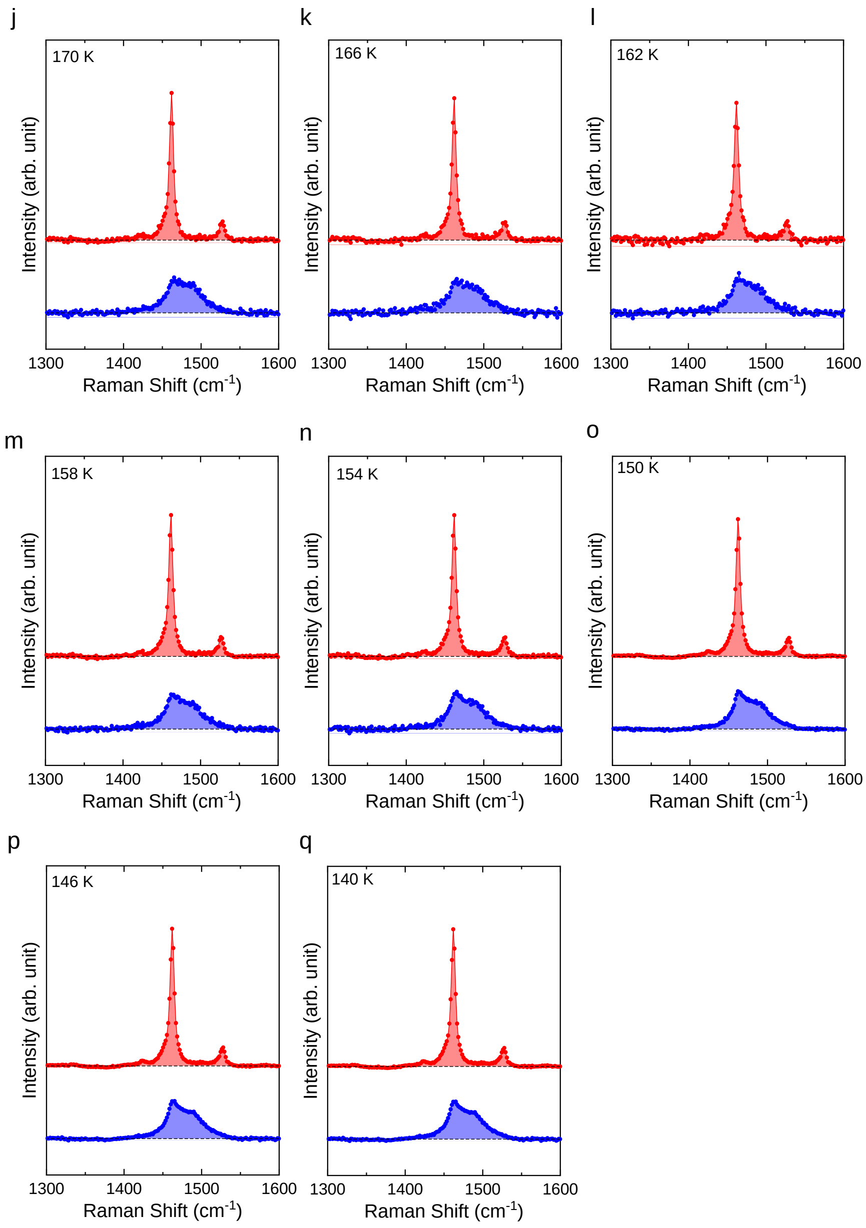

Supplementary Figure 1. Raman spectra of SCL/CG (blue) and CO (red) at all temperatures studied. a-q shows the Raman spectra of SCL/CG (blue) and CO (red) at temperatures indicated in each panel. These spectra were used in the analysis shown in Fig. 2.

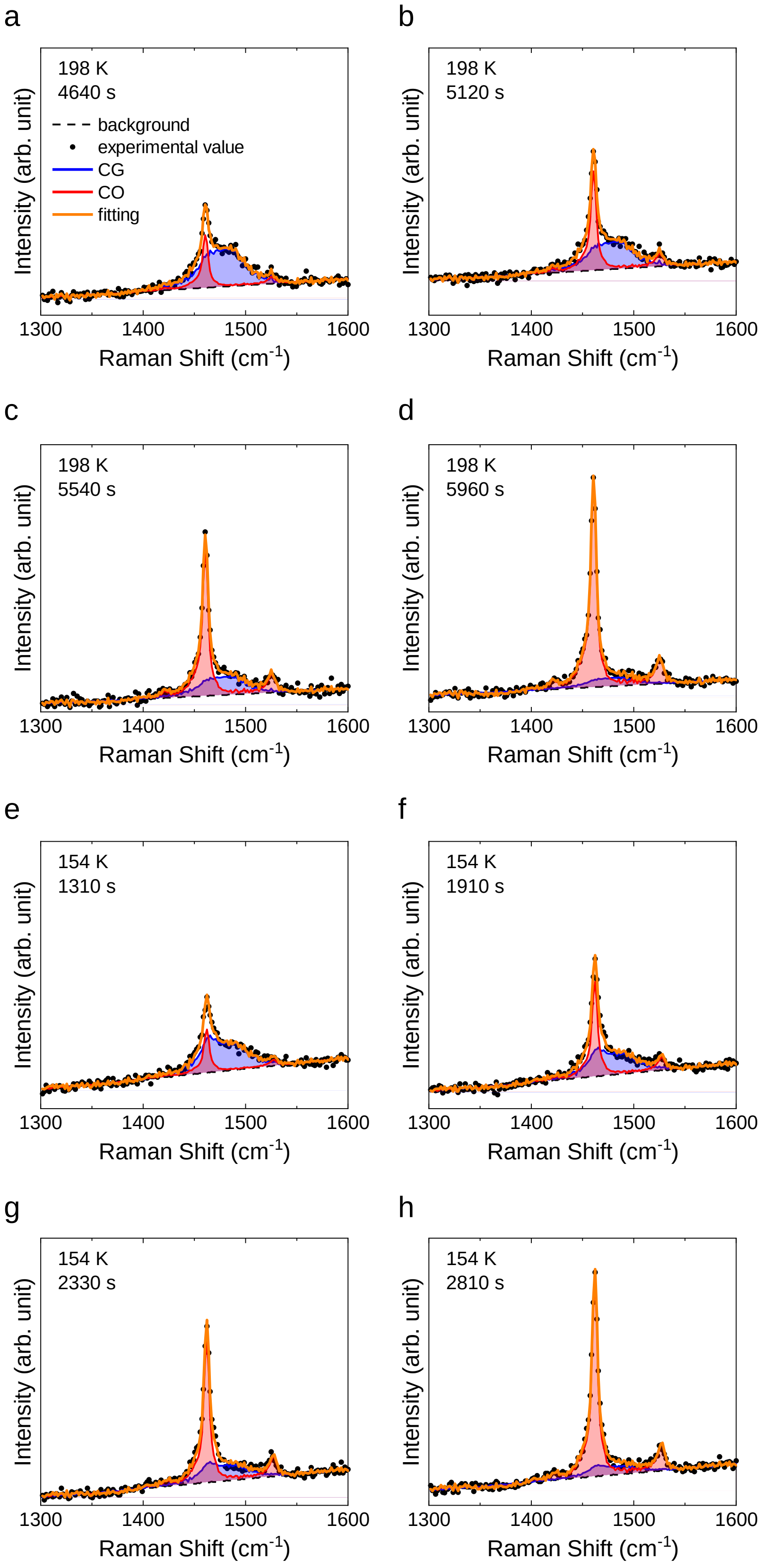

a
198 K
4640 s
background
experimental value
CG
CO
fitting
b
198 K
5120 s
c
198 K
5540 s
d
198 K
5960 s
e
154 K
1310 s
f
154 K
1910 s
g
154 K
2330 s
h
154 K
2810 s
Intensity (arb. unit)
Raman Shift (cm-1)
1300
1400
1500
1600

Supplementary Figure 2. Raw data of the Raman spectroscopy in the crystallization process. a-d show the time evolution of the Raman spectra at 198 K. **e-h** show the time evolution of the Raman spectra at 154 K. The black, red and blue lines indicate $I(\nu, t)$, $I^{\mathrm{CO}}(\nu)$ and $I^{\mathrm{CG}}(\nu)$, respectively. The black dashed lines indicate background well approximated by the form of $a\nu + b$. The orange dashed lines indicate the fitting curves.

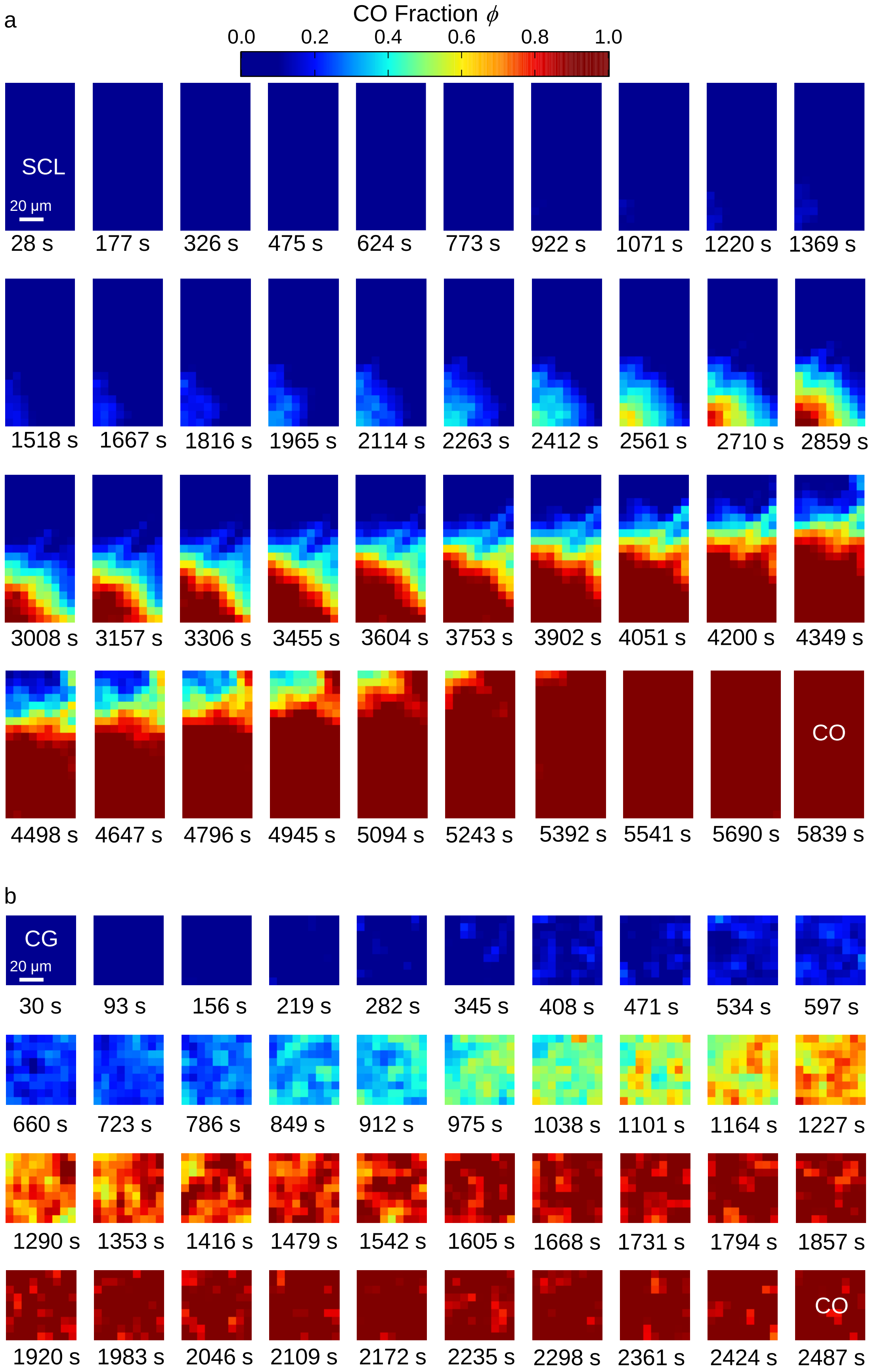
a
CO Fraction ϕ
0.0
0.2
0.4
0.6
0.8
1.0
SCL
20 µm
28 s
177 s
326 s
475 s
624 s
773 s
922 s
1071 s
1220 s
1369 s
1518 s
1667 s
1816 s
1965 s
2114 s
2263 s
2412 s
2561 s
2710 s
2859 s
3008 s
3157 s
3306 s
3455 s
3604 s
3753 s
3902 s
4051 s
4200 s
4349 s
4498 s
4647 s
4796 s
4945 s
5094 s
5243 s
5392 s
5541 s
5690 s
5839 s
CO
b
CG
20 µm
30 s
93 s
156 s
219 s
282 s
345 s
408 s
471 s
534 s
597 s
660 s
723 s
786 s
849 s
912 s
975 s
1038 s
1101 s
1164 s
1227 s
1290 s
1353 s
1416 s
1479 s
1542 s
1605 s
1668 s
1731 s
1794 s
1857 s
1920 s
1983 s
2046 s
2109 s
2172 s
2235 s
2298 s
2361 s
2424 s
2487 s
CO

Supplementary Figure 3. Time evolution of the Raman Images during isothermal crystallization. **a**, Crystallization at 195 K. **b**, Crystallization at 155 K. The scale bar indicates 20 μm.

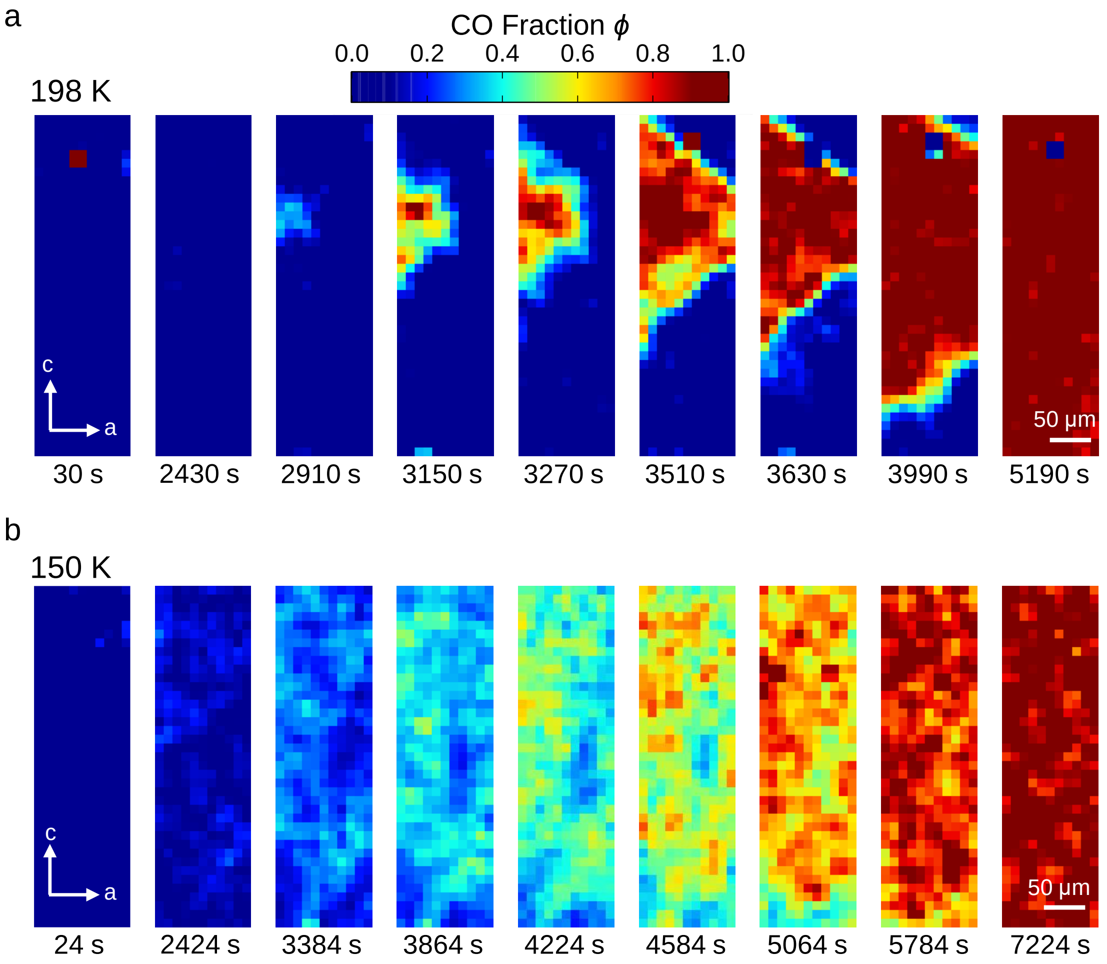

Supplementary Figure 4. Time evolution of the Raman Images during isothermal crystallization in a large area. **a**, Crystallization at 198 K. **b**, Crystallization at 150 K.

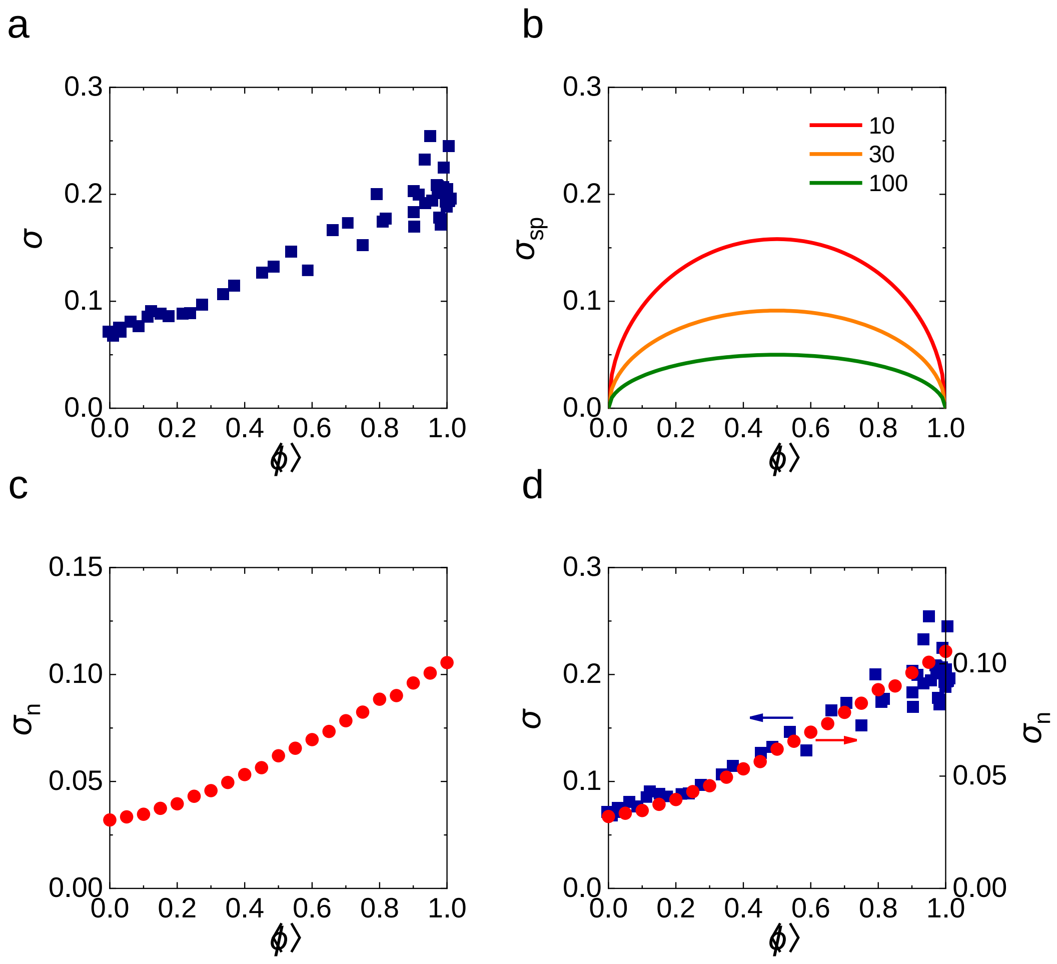

Supplementary Figure 5. Experimental and simulation values of variance of ϕ**. a**, experimental values of variance of ϕ. **b**, Theoretical variance of ϕ in the site-percolation model with 10, 30 and 100 sites in one pixel. **c**, Calculated variance of ϕ for the sum of the ideal CO and CG Raman spectra with artificial gaussian noise. **d**, Comparison of the results of a and c.

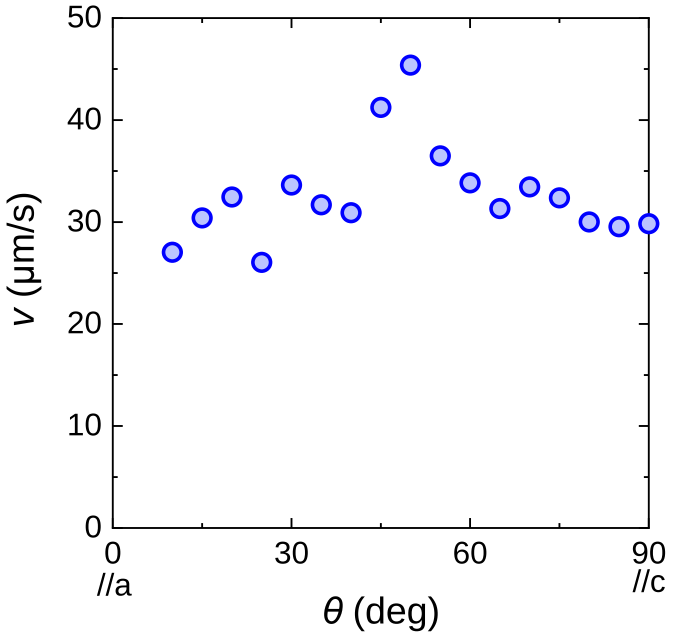

Supplementary Figure 6. Directional dependence of the growth speed.

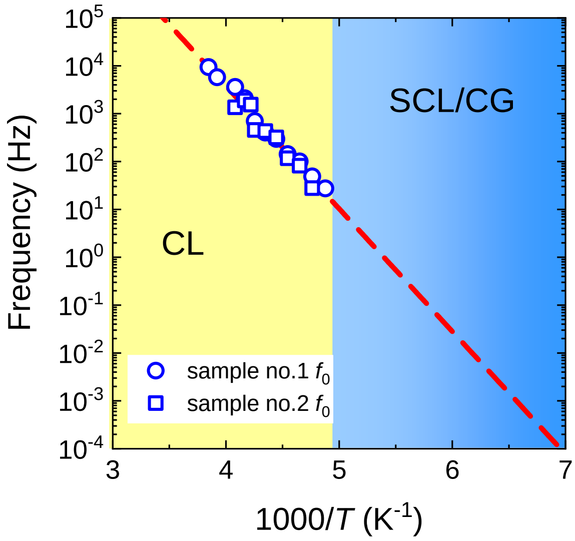

Supplementary Figure 7. Extrapolation of charge fluctuation frequency. The f_0 [$=(f_{c1}f_{c2})^{1/2}$] values in the CL phase are plotted with using the f_{c1} and f_{c2} values reported in [1]. The dashed line is a fit of Supplementary Eq. (2), which is extrapolated to the SCL/CG phase in the analysis (see main text).

Supplementary Reference

1. Kagawa, F. *et al*. Charge-cluster glass in an organic conductor. *Nat. Phys.* **9**, 419–422 (2013).
