# Supplementary material for: Spatiotemporal observation of quantum crystallization of electrons
Source: Nat Commun. 2023 Sep 26;14:6011. doi: 10.1038/s41467-023-41731-7 (PMC10522630; doi:10.1038/s41467-023-41731-7)
Supplement: Supplementary file 3 — Description of Additional Supplementary Files [file 41467_2023_41731_MOESM3_ESM.docx]

<Description of Additional Supplementary Files>

File Name: Supplementary Movie 1
Description: Time evolution of Raman image for electronic crystallization at 195 K. Blue and red colours indicate the SCL and CO regions, respectively.

File Name: Supplementary Movie 2
Description: Time evolution of Raman image for electronic crystallization at 155 K. Blue and red colours indicate the CG and CO regions, respectively.
